# Draft Genome Sequence of Francisella tularensis subsp. *holarctica* Strain H0001, Isolated from a Tularemia Patient in the Republic of Korea

**DOI:** 10.1128/MRA.00719-21

**Published:** 2022-01-27

**Authors:** Jin Sun No, Il-Hwan Kim, Chi-Hwan Choi, Jun Ho Jeon, Gi-eun Rhie

**Affiliations:** a Division of High-Risk Pathogens, Bureau of Infectious Disease Diagnosis Control, Korea Disease Control and Prevention Agency, Cheongju, Republic of Korea; University of Maryland School of Medicine

## Abstract

Francisella tularensis is the etiological agent of the zoonosis tularemia. Here, we report the draft genome sequence of F. tularensis subsp. *holarctica* H0001, which was isolated from a tularemia patient in the Republic of Korea.

## ANNOUNCEMENT

Francisella tularensis, a Gram-negative bacterium, is the causative agent of tularemia (1). Tularemia mainly occurs in Europe, northern and central Asia, and North America (2). Tularemia is caused by contact with infected animals, arthropod vectors, contaminated water and food, and inhalation of contaminated dust (3). Depending on how the bacteria enter the body, the clinical presentation of tularemia can be ulceroglandular, glandular, oculoglandular, oropharyngeal, typhoidal, or pneumonic (4). The subspecies of F. tularensis are comprised of *tularensis*, *holarctica*, *novicida*, and *mediasiatica*, with different virulences and geographic distributions (5).

In the Republic of Korea, a tularemia case was reported in 1998 (6). Before the onset of symptoms, the patient had contact with a dead rabbit and ate it. Strain H0001 was isolated by direct plating lymph node homogenates of the patient onto blood agar plates and culturing them for 5 days. The isolated colonies were then subcultured on chocolate agar plates containing cystine for 7 days at 37°C in a 5% CO_2_ incubator. The strain was confirmed as F. tularensis subsp. *holarctica* based on microbiological tests such as agglutination, direct fluorescent antibody staining using the standard serum from U.S. Centers for Disease Control and Prevention, and biochemical tests, including Gram staining, acid production, oxidase, and urease tests (6).

Genomic DNA was extracted from the strain cultured on chocolate agar at 37°C in a 5% CO_2_ incubator for 72 h using an i-genomic BYF (bacteria, yeast, fungi) DNA extraction minikit (iNtRON Biotechnology, Seongnam, Republic of Korea). The library was prepared using the Nextera DNA Flex library preparation kit (Illumina, San Diego, CA, USA). Sequencing was performed on the Illumina MiSeq instrument in 2 × 300-bp format using a MiSeq reagent kit v3 (Illumina). In total, 4,956,978 reads were obtained. The reads were filtered to remove adapter sequences and low-quality sequences using Trim Galore v0.6.1 up to a quality value of Q30. The filtered reads were assembled using the *de novo* assembly modules in CLC Genomics Workbench v20 (Qiagen, Valencia, CA, USA) with default parameters. The gaps were closed using IMAGE (7) and GapFiller (8). The assembly totaled 1,836,032 bp, with 64 contigs, an average coverage of 585×, an *N*_50_ value of 44,018 bp, and a G+C content of 32.18%. The completeness of the assembly was assessed using BUSCO v5.2.2 (9) with the data set thiotrichales_odb10, and the score was 98.2% (491 complete sets). Genome annotation was performed using the NCBI Prokaryotic Genome Annotation Pipeline (PGAP) v5.2 (10). A total of 1,675 predicted protein-coding genes were identified, along with 6 rRNAs and 34 tRNAs.

In this study, we report the draft genome sequence of F. tularensis subsp. *holarctica* H0001, which was isolated from a tularemia patient in the Republic of Korea. This draft genome sequence will serve as a reference for comparative genomics with other F. tularensis strains.

### Data availability.

The assembly has been deposited at NCBI GenBank under accession number JAJJMT000000000.1. The BioProject, BioSample, and SRA accession numbers are PRJNA735037, SAMN19554707, and SRS9132933, respectively. 
